# Noise-Induced Frequency Modifications of Tamarin Vocalizations: Implications for Noise Compensation in Nonhuman Primates

**DOI:** 10.1371/journal.pone.0130211

**Published:** 2015-06-24

**Authors:** Cara F. Hotchkin, Susan E. Parks, Daniel J. Weiss

**Affiliations:** 1 Intercollege Graduate Degree Program in Ecology, Penn State University, University Park, PA, United States of America; 2 Department of Biology, Syracuse University, Syracuse, NY, United States of America; 3 Department of Psychology and Program in Linguistics, Penn State University, University Park, PA, United States of America; University of Salamanca- Institute for Neuroscience of Castille and Leon and Medical School, SPAIN

## Abstract

Previous research suggests that nonhuman primates have limited flexibility in the frequency content of their vocalizations, particularly when compared to human speech. Consistent with this notion, several nonhuman primate species have demonstrated noise-induced changes in call amplitude and duration, with no evidence of changes to spectral content. This experiment used broad- and narrow-band noise playbacks to investigate the vocal control of two call types produced by cotton-top tamarins (*Saguinus Oedipus*). In ‘combination long calls’ (CLCs), peak fundamental frequency and the distribution of energy between low and high frequency harmonics (*spectral tilt*) changed in response to increased noise amplitude and bandwidth. In chirps, peak and maximum components of the fundamental frequency increased with increasing noise level, with no changes to spectral tilt. Other modifications included the Lombard effect and increases in chirp duration. These results provide the first evidence for noise-induced frequency changes in nonhuman primate vocalizations and suggest that future investigations of vocal plasticity in primates should include spectral parameters.

## Introduction

Noise is known to induce a variety of vocal modifications during acoustic communication in mammals, anurans, and birds, among other taxa, including changes to the amplitude, temporal, and spectral features of vocalizations (for reviews, see: Brumm and Slabbekoorn [1],Brumm and Zollinger [2]). Such changes may enable signalers to compensate for suboptimal transmission environments and increase their chances of successful communication [1,3,4]. While some vocally flexible species are able to control the acoustic structure of their vocalizations during normal communication [5], most studies of the Lombard effect indicate that it is an involuntary response to increases in background noise [6]. Human speakers struggle to suppress the Lombard effect in speech, even using visual feedback [7], and neurophysiological studies indicate that the basic neural circuitry that generates the Lombard response can be found in the brainstem [8,9].

In human speech, the Lombard effect has been linked to temporal and spectral modifications, which have also been detected in other vertebrate species [1,4,10]. Current hypotheses propose that spectral modifications may be non-adaptive biomechanical or psychophysical byproducts caused by increased air pressure within the vocal tract during increased vocal effort [3,11–13]. A second alternative is that the production of higher frequency vocalizations may be easier to achieve at higher amplitudes. Masking release via frequency shifts may therefore include increases in call amplitude by default [14,15]. Previous research has demonstrated that spectral shifts in human speech are dependent on the spectral content of the noise [6]. However, recent data from noise exposure experiments with avian and mammalian taxa show that some vertebrate species are capable of immediate and independent changes to the amplitude and spectral characteristics of vocalizations during increased noise [16–18], suggesting that spectral modifications may serve a communicative function during increased noise. This view accords with the recent suggestion that multiple levels of vocal control may be active during Lombard vocalizations. Correspondingly, vocal modifications due to noise could involve a complex array of neural structures extending beyond those involved in brain stem reflexes [19].

Among non-human primates, noise-induced vocal modifications have been studied in common marmosets (*Callithrix jacchus*), gray mouse lemurs (*Microcebus murinus*), two species of macaques (*Macaca fascicularis* and *M*. *nemestrina*), and cotton-top tamarins (*Saguinus oedipus*) [20–25]. Interestingly, while all of these species have been found to modify their call amplitude during noise, there is very limited evidence for noise-induced spectral modifications. While Brumm et al. [25] did not investigate spectral changes in marmoset calls, cotton-top tamarins showed no evidence of consistent changes in average fundamental frequency (F_0_) despite a significant Lombard effect [20]. To date, the only evidence for spectral changes in response to noise comes from a study of the gray mouse lemur that reported some reduction in the variability of the fundamental frequency of calls during noise [23]. The overall lack of vocal flexibility found in the spectral domain is reminiscent of the long-standing notion that nonhuman primates are capable of controlling acoustic parameters that can be modulated by changes in exhalation, such as loudness and duration, but lack control over spectral features that may require more nuanced control over the vocal apparatus [5]. Nevertheless, as there is a relative paucity of studies on vocal modifications in response to noise in primates, it remains an open question as to whether they are capable of modifying the spectral features of their calls in this context.

One noise-induced spectral modification that has been studied almost exclusively in human speech is spectral tilt, a measure of the relative energy in low and high frequency portions of a signal [4,26–28]. During increased noise, energy shifts from low frequency portions of the signal to higher frequency bands (referred to as a decrease in spectral tilt), potentially removing some of the signal from masking noise under certain noise conditions [26,29]. Additionally, there is evidence that some mammals may be capable of increasing spectral tilt (more energy at lower frequencies) when exposed to high frequency noise, though this has yet to be empirically tested [30]. For humans, changes in spectral tilt appear to be closely related to the Lombard effect [3,11–13], and changes to spectral tilt have been shown to increase the intelligibility of speech during increased noise [26], possibly providing some communicative advantage independent of the Lombard effect. While two nonhuman species have shown evidence for changes to spectral tilt during increased noise [30,31], the only study to have investigated this phenomenon in a nonhuman primate species (gray mouse lemurs) found no evidence for its presence [23].

Cotton-top tamarins are New World monkeys native to the rainforests of Colombia [32], with an extensive vocal repertoire [33], including a stereotyped vocalization known as the ‘combination long call’ (CLC), which consists of “chirp” and “whistle” components, and is presumed to function as a long-distance contact call [34–38]. This call type is often produced in response to other individuals producing CLCs (‘antiphonal calling’). Perceptually salient components of this call type have been identified using both production and perceptual measures [39–41]. Chirp vocalizations are known to be produced in a variety of contexts, including alarm calls, food calls, and responses to novel stimuli (e.g. Cleveland and Snowdon [33]). Chirps variants often significantly overlap in their acoustic properties, although there is evidence suggesting that tamarins may nonetheless pick up on relevant acoustic features that differentiate the calls (e.g., Bauers & Snowdon, [42]).

Evidence for independent functions of the Lombard effect and noise-induced spectral modifications is intriguing and raises the possibility of separate communicative functions for amplitude and frequency shifts, particularly under the conditions of noise with varying frequency spectra. The broad vocal repertoire of the cotton-top tamarin (documented in [33]), as well as their need to communicate across distances, make them an ideal species to further investigate this effect. While the previous study with tamarins found no change in the fundamental frequency of CLCs during increased noise [21], there have been no direct tests of the effects of different noise frequencies on vocal structure in this species, and no examination of potential changes to spectral tilt. If noise-induced vocal modifications are dependent on the frequency of the noise, as they are in humans [6,13], we would predict differential changes in frequency content of calls based on spectral overlap with noise stimuli. This study therefore used playbacks of both broad- and narrowband noise to investigate cotton-top tamarins’ control of vocalization frequency content and spectral tilt. We chose to investigate two call types. In keeping with the previous study [21], we analyzed CLCs and we also included shorter chirp vocalizations. These chirps would best be characterized as Type G chirps, as they were produced during periods of relaxed exploration (see [33]).

## Materials and Methods

### Animal Care

The tamarins used for this study were housed at The Pennsylvania State University, University Park, PA in a single colony room that allowed unlimited acoustic contact by all colony members and limited visual contacts with animals in neighboring home cages. The colony was housed in mated pairs with the exception of a single male housed alone due to the death of its mate. The monkeys were born at the New England Regional Primate Research Center in Southborough, MA, and were brought to Penn State in June 2005. They had ad libitum access to water and were not weight restricted. Home cages contained branches and small toys for enrichment; these items are moved and rotated on a regular basis. Seven tamarins participated in this study. However, two of the subjects did not vocalize during trials and were excluded from analysis, yielding a sample size of five subjects (3 males and 2 females), none of whom were cagemates. Use and care of the tamarins conformed to the rules and regulations of the IACUC at the Pennsylvania State University who granted approval for this study (protocol # 43831). No subjects were sacrificed during this study.

### Data collection

**Fig 1:**
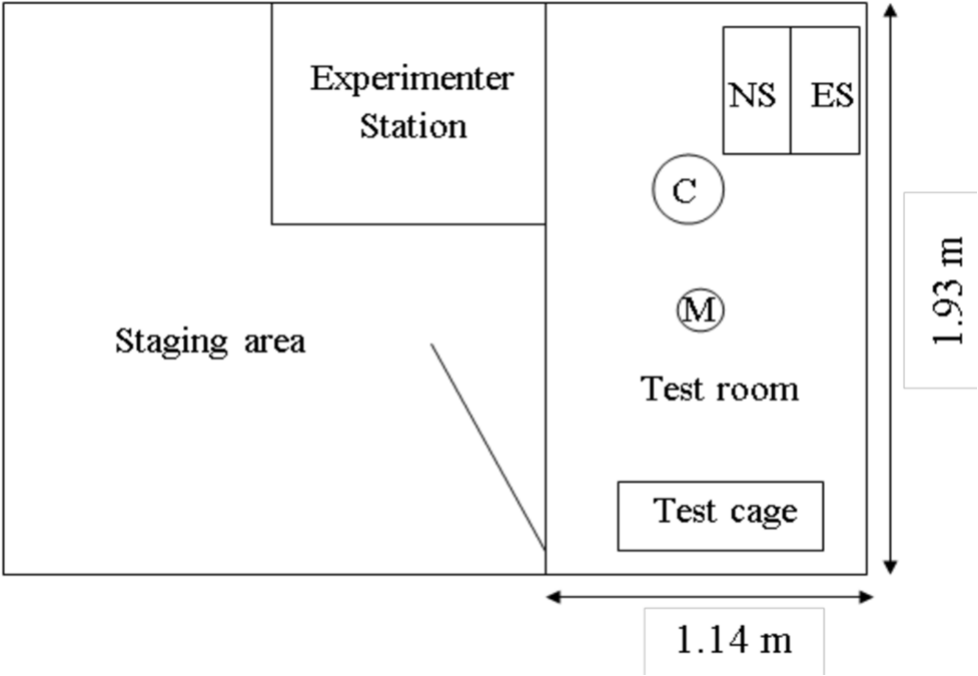
Diagram of experimental setup. Letters indicate equipment placement; M = microphone; C = video camera; NS = speaker presenting noise stimulus; ES = speaker presenting elicitation stimulus.

Data were collected between November 2011 and March 2012. The testing area consisted of a soundproof testing room (Acoustic Systems, customized chamber) lined with acoustic foam to reduce reverberation (Fig 1). Acoustic stimuli were projected into the chamber through a two-channel power amplifier connected to speakers mounted 1.5m above the floor inside the testing room. The sound projection system had a flat frequency response from 63 Hz—30 kHz (± 3 dB). Noise stimuli were played through a Marantz PMD620 recorder connected to one channel of the amplifier. The second amplifier channel was used to present call elicitation stimuli. The test cage (0.6 x 0.3 x 0.6 m; bottom and front made of steel mesh; all other sides made of plastic) was positioned at the opposite end of the chamber from the speakers.

The subjects’ behavior and positioning within the test cage were monitored in real time via streaming video. If the subject showed signs of distress, such as rapid movements within the cage or alarm vocalizations, the session was aborted and re-run on another day. Only five (Mulva – 2; Susan – 2; Bart – 1) trials were aborted due to distress behaviors. Acoustic data were recorded with a calibrated Earthworks M30 omnidirectional microphone mounted 0.3 m from the floor, facing the front (steel mesh) side of the test cage and Edirol R40 Pro data acquisition system with a sampling rate of 48 kHz. Noise within the test cage was recorded before the beginning of data collection; levels within the cage never exceeded 70 dB re 20 μPa rms; stimulus amplitudes ranged from 2 to 22 dB above ambient sound levels (44 to 64 dB re 20 μPa rms; S1 Table). Examination of the video recordings confirmed that subjects vocalized while facing the microphone equally during control and treatment trials.

#### Playback stimuli

**Fig 2:**
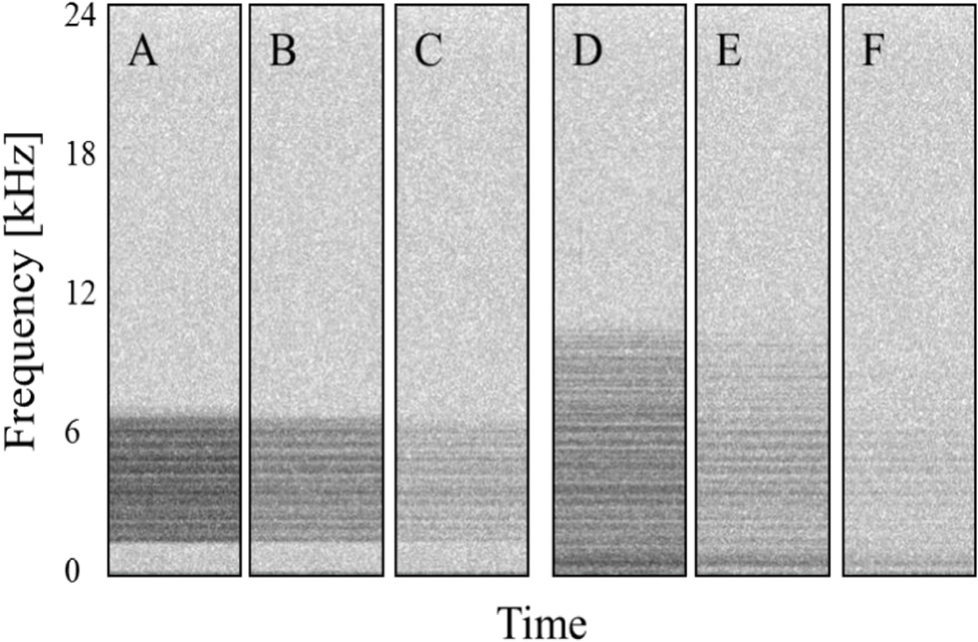
Noise stimuli. Spectrograms (1024 point Hamming window, 75% overlap, 11.7 Hz frequency resolution) of noise playback stimuli recorded during trials. Treatments A–C had a bandwidth of 5 kHz. Treatments D–F had a 10 kHz bandwidth. Harmonic structure is due to frequency response of the playback system.

White noise stimuli were generated in Adobe Audition (sampling rate: 44.1 kHz), and band-pass filtered to create 6 noise bandwidth and level combinations (Fig 2). Noise bandwidths and frequency ranges were selected to target the masking noise at the perceptually important second harmonic of combination long calls (approximately 4 kHz; see [41]). Broadband stimuli contained energy between 100 Hz and 10 kHz; narrowband stimuli ranged from 1.5 kHz to 6.5 kHz. In the narrowband treatment, the first 3 to 4 harmonics of the call were masked, while in the broadband treatment, masking noise overlapped the first 6 harmonics.

All subjects produced spontaneous vocalizations, but to increase sample size production of CLCs was encouraged with playbacks of CLCs from an unfamiliar adult female tamarin (‘elicitation stimuli’) [34,35,39]. To elicit antiphonal calls from the test subject, three exemplars from the same female were played in random order at approximately 30s intervals during each trial; the same three exemplars were used for all subjects over the course of the experiment. There were no significant acoustic differences in the duration, minimum, or peak frequencies of calls produced spontaneously and those produced after exposure to elicitation stimuli (S2 and S3 Tables).

#### Playback trials

A maximum of one session was conducted with each subject on each testing day, and no subject experienced more than two sessions per week. Each session consisted of two trials; control and treatment trials were presented in random order (max 12 min/trial) with a rest period between trials (15–60 min.). Order of treatments was also randomized within and between subjects. In control trials, a playback speaker was active but no noise was transmitted. During treatment trials, the playback began before the subject entered the testing area and ceased at the end of the test period. All trials began with a two-minute acclimation period to allow the subject to adjust to the test room and account for any possible lag between noise exposure and the onset of vocal modifications. Upon completion of the second trial, the subject was returned to its home cage.

### Data analyses

Acoustic analyses were performed with Raven 1.4 [43] and Matlab 7 [44].

**Fig 3:**
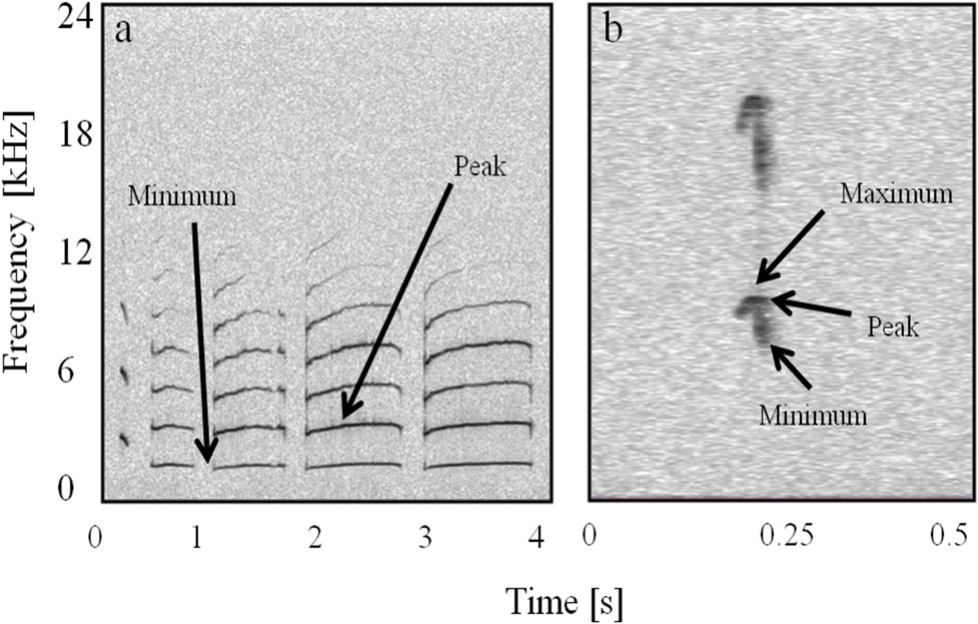
Spectrograms of CLC (a) and chirp (b) vocalizations with measured frequency characteristics indicated. This CLC consists of one chirp and four whistle syllables. All measurements of CLCs were made on the call as a whole and the individual syllables within the call (not shown); measurements of chirps were made from the fundamental frequency (first harmonic). Note that peak frequency measurements for all syllables, fundamental frequencies, and whole calls were taken automatically from the selection spectrum view in Raven 1.4 (not shown).

Measured parameters differed slightly for the two call types due to differences in overall call structure (Fig 3A). For CLCs, measured parameters included amplitude, duration, minimum (lowest) and peak (highest amplitude) frequencies of the entire call, minimum and peak frequency of the fundamental contour, and spectral tilt. Frequency measurements were taken from the power spectrum of the entire call. Peak frequency was detected automatically, while minimum and maximum frequencies were measured as the minimum and maximum inflection points at either end of the call spectrum. Fundamental frequency measurements used the power spectrum of the fundamental frequency only. Measurements were taken from the call as a whole and from each syllable within a call. Duration of whole CLC vocalizations included inter-syllable pauses, which were excluded when the individual syllable durations were measured. For non-CLC chirps, amplitude, duration, frequency (minimum, peak and maximum), and spectral tilt were measured. All frequency measurements for this call type were performed on the fundamental frequency contour (Fig 3B).

Custom Matlab scripts were used to determine noise amplitude and vocalization source levels. Noise amplitude for each trial was calculated from a 5s clip with no vocalizations and no cage noise. Received level of vocalizations was calculated as the square root of the mean-squared noise amplitude subtracted from the mean-squared amplitude of the vocalization clip. Call source level for each trial was generated by averaging the calculated root mean-squared (rms) source levels for all calls in a trial and transforming into dB re 20 μPa.

Spectral tilt was measured as the ratio of the amount of energy in the high frequency harmonics of the vocalization to the energy in the call’s fundamental frequency. Custom Matlab scripts were used to generate an averaged power spectral density for a vocalization, subtract the noise spectrum, and find peaks in the call’s power spectrum (harmonics). The resulting spectral density value for each harmonic was divided by the value for the fundamental frequency to give a ratio of energy over the entire frequency bandwidth of the vocalization. For CLCs, up to eight harmonics were recorded, while high fundamental frequencies of chirp vocalizations and the sampling rate of the recorder limited the number of chirp harmonics available for measurement to two. Statistical analysis of spectral tilt was limited to a single harmonic in both call types (CLCs–third harmonic; chirps–second harmonic) in order to maintain consistency in the analysis of both call types.

Statistical analyses were performed in SAS version 9.2. Analysis of covariance (ANCOVA) models were generated to determine the effects and interactions of noise level and bandwidth on source level, peak frequency, minimum frequency, duration, and spectral tilt of both call types. In all models, noise level was treated as a continuous variable and subject identity was included as a random effect.

## Results

Five subjects produced vocalizations during this study. While all five subjects produced both types of calls, the numbers of calls produced varied between animals and not all subjects produced sufficient quantities of both types of vocalizations for analysis. Of the five, three (Bart, Jerry, and Mulva) reliably produced CLCs (N = 159; Table 1) and three (Bart, Milhouse and Susan) produced spontaneous non-CLC chirps (N = 833; Table 1). Call types and associated measurements are shown in Fig 3. Vocalization type appeared to affect the types of vocal modifications observed, with different suites of modifications detected in CLCs and chirps. Inter- and intra-individual variability was high in all trials. Statistical results are summarized in Table 2.

**Table 1:** Numbers of CLCs and chirps produced by each subject during the “test” periods summed over all six noise types (total of 12 trials per subject).

		**Bart**	**Jerry**	**Mulva**	**Total**
**CLCs**	Control	23	18	33	74
	Treatment	24	30	31	85
		**Bart**	**Milhouse**	**Susan**	**Total**
**Chirps**	Control	91	245	156	492
	Treatment	77	185	79	341

Note that calls produced during pre-test acclimation periods are not included in this table.

**Table 2:** Statistical Results for repeated measures ANCOVA for all variables measured for CLCs and chirps displayed by noise level [NL] and bandwidth [BW]).

		NL		BW		NL*BW	
		**F**_**1,30**_	**p**	**F**_**1,30**_	**p**	**F**_**1,30**_	**p**
**CLCs**	Source level (whole call)	33.64	**0.01**	1.86	0.18	3.13	*0*.*09*
	Source level (whistles)	11.21	**0.01**	1.28	0.27	2.1	0.16
	Duration (whole call)	2.42	0.13	0.06	0.8	0.02	0.88
	Duration (whistles)	0.41	0.52	0.41	0.43	0.42	0.52
	Peak fundamental frequency	4.99	**0.03**	2.19	0.15	3.45	*0*.*07*
	Minimum fundamental frequency	2.18	0.15	8.03	**0.01**	8.69	**0.01**
	Peak frequency (whole call)	4.8	**0.04**	0.06	0.81	0.13	0.72
	Peak frequency (whistles)	21.07	**0.01**	0.01	0.94	0.01	0.94
	Spectral tilt	19.98	**0.01**	2.37	0.13	2.88	0.1
		**F**_**1,26**_	**p**	**F**_**1,26**_	**p**	**F**_**1,26**_	**p**
**Chirps**	Source level	5.89	**0.02**	0	0.996	0.03	0.85
	Duration	9.41	**0.01**	0.12	0.73	0.05	0.83
	Peak frequency	6.17	**0.02**	0.8	0.38	0.61	0.44
	Maximum frequency	5.83	**0.02**	0.68	0.42	0.55	0.47
	Minimum frequency	2.95	0.1	0	0.99	0.02	0.88
	Spectral tilt	2.76	0.11	0.42	0.52	0.57	0.46

Noise playbacks included broadband (0.1–10 kHz) and narrowband (1.5–6.5 kHz) stimuli (Fig 2), at a range of amplitudes. Ambient noise (0.1–24 kHz) during control sessions was approximately 42 dB re 20 μPa rms, and the highest exposure level was approximately 66 dB re 20 μPa rms during treatment A (narrowband, high amplitude).

### The Lombard Effect

**Fig 4:**
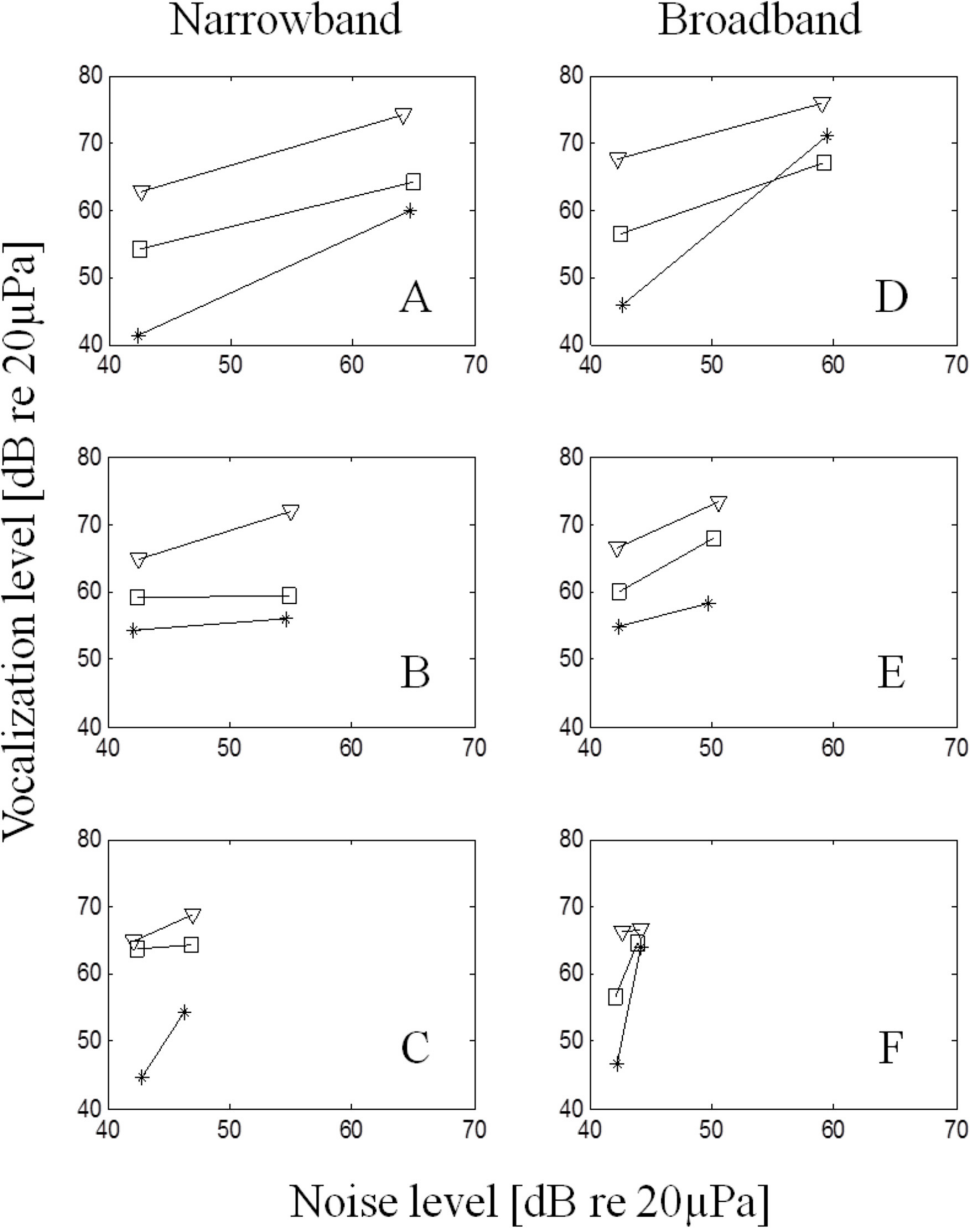
Average CLC source levels vs. noise level for all 6 noise bandwidth/level combinations. Treatments A, B, and C had 5 kHz bandwidth; treatments D, E, and F had 10 kHz bandwidth. The top, middle, and bottom rows indicate high, medium, and low noise amplitudes, respectively. Panels are arranged by noise level (rows) and bandwidth (columns). Triangles, squares, and stars represent Mulva, Jerry, and Bart, respectively. Note that call source level never decreases between control (42 dB re 20 μPa noise level) and treatment trials.

The Lombard effect was observed for both call types (Table 2). For combination long calls (CLCs), a repeated-measures ANCOVA model revealed a main effect of noise level for both whole calls (F_1,30_ = 33.64; p < 0.01) and whistle syllables (F_1,30_ = 11.21; p < 0.01). While there was no main effect of noise bandwidth, the interaction of noise level and bandwidth trended towards significance (p = 0.07), with subjects increasing vocal amplitude slightly more during broadband than narrow-band noise (Fig 4), despite overall lower energy in the broadband stimuli (S3 Table).

Amplitude of chirp vocalizations was also significantly higher during increased noise (F_1,26_ = 5.89; p = 0.02), but the overall magnitude of the effect was smaller for chirps than for CLCs (S4 Table).

### Temporal Modifications

No changes to the duration of CLCs were detected in either whole CLC vocalizations (F_1,30_ = 2.42; p = 0.13) or whistle syllables (F_1,30_ = 0.41; p = 0.52). Duration of chirps increased significantly with increased noise amplitude (F_1,26_ = 9.41; p<0.01). The average change in chirp duration was approximately 5.5 ms; average chirp durations for all three individuals are given in Table 3. There was no main effect of bandwidth and no amplitude-bandwidth interaction (Table 2).

**Table 3:** Average chirp duration (ms).

	Control	A	B	C	D	E	F
**Bart**	53.7	Na	64.2	54.2	58.7	59.3	na
**Milhouse**	60.0	61.1	59.5	58.8	65.7	76.3	64.9
**Susan**	60.6	76.3	59.9	60.1	67.4	65.0	74.9

Control values are were averaged over all six control trials for each subject; treatment values were averages of each chirp produced by a subject during the named treatment trial. Bart did not produce chirps during treatments A and F.

### Spectral Modifications

Noise level and bandwidth both affected the spectral properties of CLCs (Table 2). Significant increases were found in the peak frequency of the fundamental harmonic of whole CLCs in relation to noise level (F_1,30_ = 4.99; p = 0.03). The interaction between noise level and bandwidth approached significance (p = 0.07; Table 2) and may merit further investigation.

The minimum frequency of the first harmonic decreased during noise playbacks. While there was no significant effect of noise level (F_1,30_ = 2.18; p = 0.15), a significant interaction of noise level and bandwidth and a main effect of bandwidth were detected (F_1,30_ = 8.69; p < 0.01; Table 2). Greater decreases in minimum frequency were observed in response to broadband noise than to narrowband noise (Table 4). There was also a significant increase in peak frequency of whole CLCs (F_1,30_ = 4.8; p = 0.04) and of whistle syllables (F_1,30_ = 21.07, p < 0.01) during increased noise amplitude.

**Table 4:** Minimum fundamental frequency of CLCs and change with each noise type.

Subject	Treatment	Control minimum fundamental frequency [Hz]	0030	Δ Minimum fundamental frequency [Hz]
**Bart**	**A**	1511	1564	53
	**B**	1393	1507	114
	**C**	1423	1499	77
	**D**	1553	1497	-57
	**E**	1568	1557	-11
	**F**	1478	1409	-69
**Jerry**	**A**	1579	2508	929
	**B**	1488	1496	9
	**C**	1536	1505	-30
	**D**	1653	1418	-236
	**E**	1489	1444	-45
	**F**	1693	1545	-148
**Mulva**	**A**	1653	1761	107
	**B**	1716	1656	-59
	**C**	1564	1631	67
	**D**	1674	1618	-56
	**E**	1671	1597	-74
	**F**	1649	1659	11

**Fig 5:**
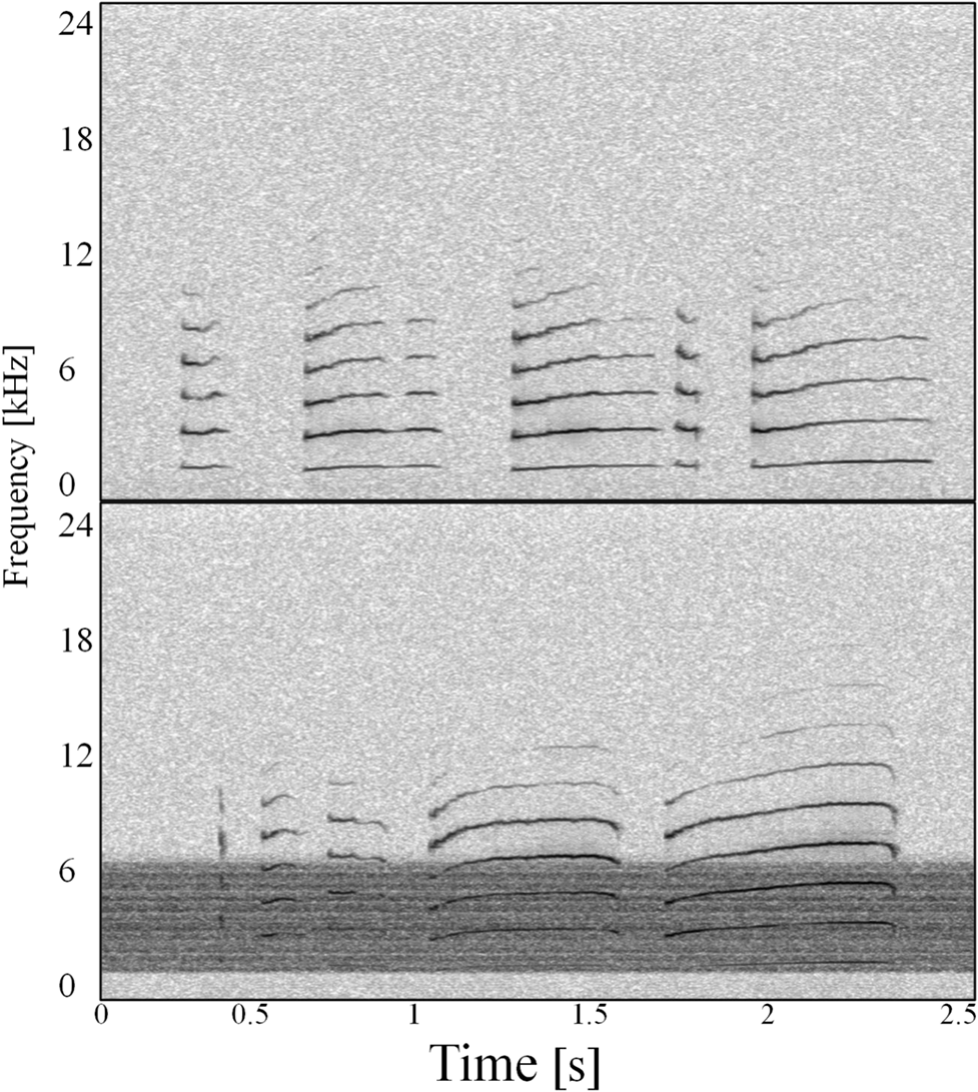
Representative CLCs produced by subject Mulva during a) control and b) treatment A trials illustrating changes to spectral tilt. All whistles from a) have strong fundamental frequencies and maximum energy in the 2^nd^ harmonic, while in b) the first whistle has a very faint fundamental frequency at approximately 2 kHz, and peak frequencies for all whistles occur in the 4^th^ harmonics. Reduced energy in the fundamental frequency is also apparent in the second and third whistles (spectrogram parameters: 1024 point Hamming window, 75% overlap, 11.7 Hz frequency resolution).

**Fig 6:**
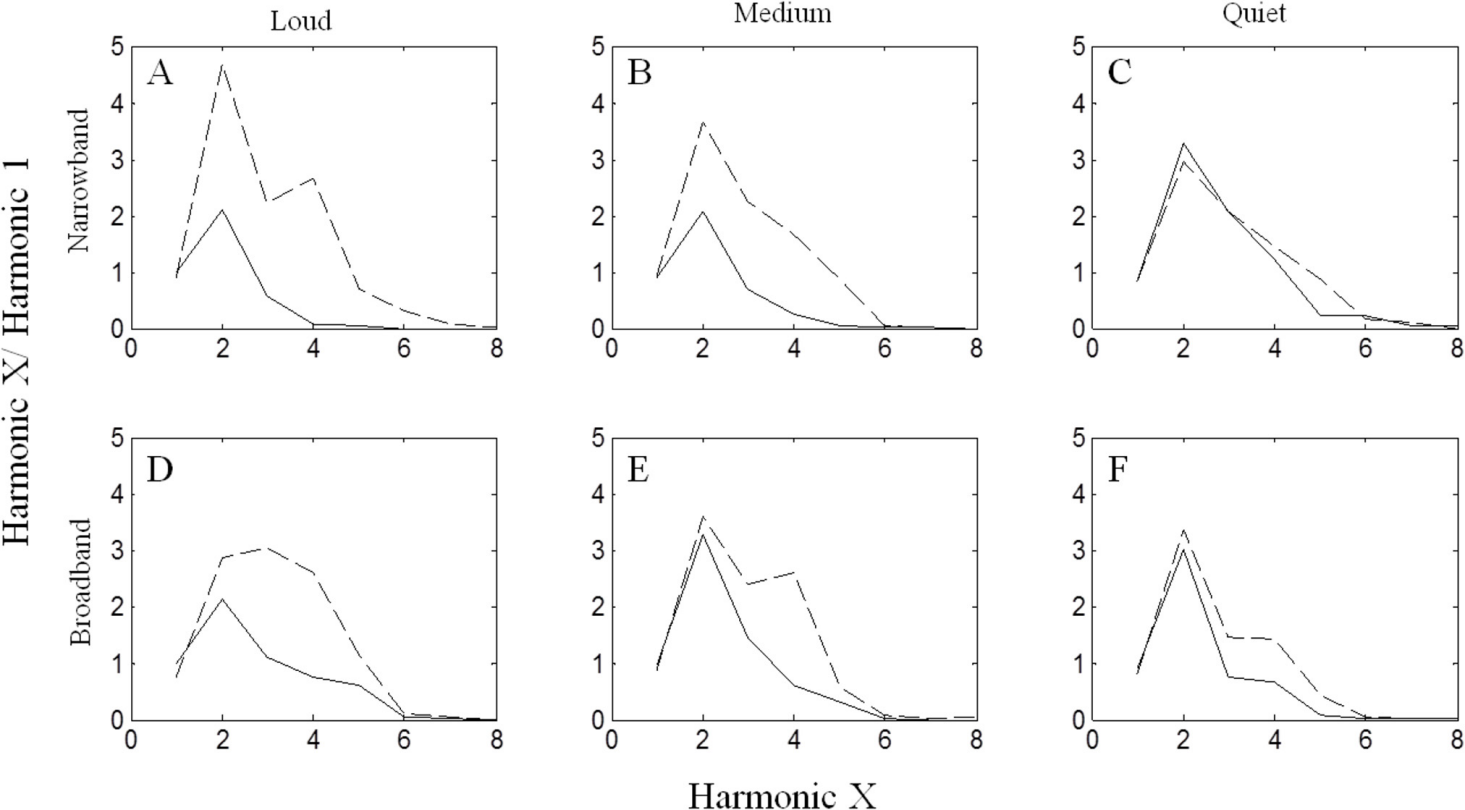
Change in spectral tilt for all 6 noise bandwidth/level combinations. Solid lines indicate control trial values averaged over all animals; dashed lines indicate treatment trial values. Treatments A, B, and C had 5 kHz bandwidth; treatments D, E, and F had 10 kHz bandwidth. The left, middle, and right columns indicate high, medium, and low noise amplitudes, respectively. Panels are arranged by noise level (columns) and bandwidth (rows).

Changes to peak frequency of whistle syllables could not be entirely accounted for by changes to the fundamental frequency of CLCs. Instead, changes in peak frequency appear to be the result of a combination of shifts in fundamental frequency and changes to spectral tilt (Fig 5). Power spectral density ratios of the third harmonic to the fundamental frequency indicated significant increases in energy at higher call frequencies during increased noise levels (F_1,30_ = 19.98; p<0.01). Spectral tilt changes were apparent for all 3 CLC-producing subjects and were strongest in the two loudest treatments (A and D) (Fig 6). In control sessions, the second harmonic generally contained the greatest amount of energy, with a ratio of 2 to 3 times the energy in the fundamental frequency. During treatment sessions, the second harmonic was still often the peak frequency, but more energy was also found up to the fifth and sixth harmonics. In trials with lower noise levels (treatments C and F), there was no difference in spectral tilt between control and treatment sessions.

Frequency parameters of chirp vocalizations also shifted upward during increased noise. There were significant increases in peak (F_1,26_ = 6.17; p = 0.02) and maximum (F_1,26_ = 5.83; p = 0.02) frequencies. There was no change in minimum frequency (F_1,26_ = 2.95; p = 0.1). Spectral tilt of chirp vocalizations (ratio of second harmonic to fundamental frequency) did not change with noise level or bandwidth (F_1,26_ = 2.76; p = 0.11).

## Discussion

The tamarins in this study demonstrated significant plasticity in vocal production for two call types, exhibiting the Lombard effect, an increase in chirp duration, and multiple changes to the frequency parameters of both call types under different conditions of spectral overlap. While the Lombard effect and other noise-induced vocal modifications were observed simultaneously in this experiment, the differences in response between whistles and chirps and with other studies of vocal compensation [20,23,25] indicate greater malleability of vocal modifications to noise in nonhuman primates. Specifically, to the best of our knowledge, these results represent the first empirical report of a change in spectral tilt in any nonhuman species, and demonstrate that short-term noise induced spectral modification in primate vocalizations is not restricted to the range of the fundamental frequency [23]. Despite having access to a restricted sample size, our results can be interpreted as an existence proof that nonhuman primates are capable of modifying the spectral content of their calls in response to noise and strongly suggest the need for additional studies with larger samples and a broad range of species, including those who share an even closer evolutionary relationship with humans.

All subjects in this study produced both types of vocalizations, although most individuals produced only CLCs or non-CLC chirps in sufficient quantities for analysis. There does not appear to be any sex- or age-related difference in call production, since all animals were adults of approximately the same age and call production was split across sexes. The individual that produced both call types in sufficient quantity for analysis was male. Additionally, all subjects were exposed to the same call stimuli from an unfamiliar adult female tamarin. It is possible that differences in call production in our subjects were due to differences in perceived social ranking [1, 32], but this analysis was not possible with our data, and this possibility should be examined in future work. A previous study of vocal modifications in cotton-top tamarins found no significant changes to the average fundamental frequency of CLCs during increased noise [21]. Two factors may have contributed to the discrepancy between this study and the present one. First, measurements taken in this experiment (minimum and peak fundamental frequencies) differed from those in the previous study (average fundamental frequency). Further, the previous study used a single noise type (broadband), and did not manipulate the frequency content of the noise. Consistent with our findings that noise bandwidth affected the minimum frequency of CLCs, studies of noise-induced vocal modifications in human speech and nonhuman primates indicate that the frequency content of the noise is perceptually salient [4,6,13,24]. Changes to the frequency content of the noise stimulus may have induced larger or more consistent changes during this experiment, and the differences in measurement methodology may have also increased the probability of detecting such changes.

The previous study also found an increase in duration of CLC syllables, which was not evident in our results. However, it is possible that that within- and between-subject variability may have masked any changes to call duration that could become evident with a larger sample size. Such differences emphasize the overall variability of noise compensation responses, even within members of the same species exposed to similar types of noise. Therefore, future work must endeavor to further establish the conditions that elicit modification to temporal and spectral parameters.

As noted above, acoustic modifications also differed by call type. CLC structure was influenced by both noise amplitude and bandwidth, with different effects in the broad- and narrowband noise conditions. For chirps, noise amplitude strongly influenced amplitude, duration, as well as maximum and peak fundamental frequencies, but there was no change in overall call structure as a result of noise bandwidth, possibly due to the minimal spectral overlap between chirps and either noise stimulus. While consistent with the upward spread of masking [1], the absence of changes to the spectral content of chirps differs from Hage et al.’s [16] finding that vocalization frequencies are modified even when calls are not directly overlapped by noise. However, the differences in study design and in auditory specialization and vocal flexibility between echolocating bats and nonhuman primates may be at the root of the discrepancies between these studies.

Differences in vocal modifications between the two call types may also be impacted by the call structure. Longer vocalizations, like CLCs, may benefit more from amplitude and frequency changes than from changes to duration. However, auditory processing is not linear, and shorter sounds may experience gain from increases in duration. This is known as temporal summation [45,46]. The increased gain from the combination of amplitude and duration increases may therefore explain the relatively low-amplitude Lombard effect observed in the non-CLC chirps when compared with the CLCs.

Interestingly, while the Lombard effect and changes to fundamental frequency components were observed in chirps, there were no changes to spectral tilt in this call type. Our recording system was not capable of capturing the whole range of chirp harmonics, making it impossible to determine if call energy shifted to frequency bands above 24 kHz. However, the lack of any shift in energy to the second harmonic could indicate that the tamarins are capable of independently modifying spectral tilt and call amplitude when there is minimal frequency overlap between noise and vocalizations. Changes to spectral tilt increase the intelligibility of human speech during increased noise [4,47], but is not clear whether it serves the same communicative function in nonhumans. Given the current controversy over the linkage between vocalization amplitude and spectral modifications [4,12,16,17], it will be important for future work to clarify the effect of spectral overlap on changes to spectral tilt, and to investigate the effect of changes to detectability and intelligibility of nonhuman animals’ vocalizations.

In summary, our results represent an existence proof for short-term modification of spectral structure of vocalizations by a non-human primate, and the first demonstrated change in spectral tilt during increased noise in any non-human species. Despite a limited sample size, our findings strongly indicate that non-human primates have the ability to rapidly modify the spectral and temporal characteristics of calls in response to noise. The extent to which this phenomenon impacts the long-standing argument regarding the lack of vocal control in nonhuman primates is still subject for debate. On the one hand, Owren et al. [48] have argued that Lombard effects are mediated at the brainstem level, and as such, do not meaningfully contribute to the discussion of the evolution of complex vocal flexibility in humans. However, it has recently been noted that while frequency modulations in response to noise may not result in the production of completely novel calls, the shifting of frequency parameters may nonetheless serve as a precursor to more sophisticated types of vocal learning [49]. Further, it has been suggested that multiple levels of vocal control may be active during Lombard vocalizations and could involve a complex array of neural structures extending beyond brain stem reflexes [19]. The data from our study do not adjudicate between these two positions, but do represent further evidence for continuity in vocal production systems between nonhuman primates and humans (e.g., Ghazanfar et al. [50]). Independent modifications to call frequency and amplitude by our subjects also supports the hypothesis that spectral modifications are not simple byproducts of increases in vocal effort [12], but may serve an adaptive communicative function during periods of increased noise.

## Supporting Information

S1 Text Expanded methods and additional analyses. (DOCX)

S1 Table Noise levels (dB re 20 μPa rms) measured during all trial sessions. Averaged over all subjects (N = 5).
(DOCX)

S2 Table Comparison of average minimum and peak frequencies from whole CLCs with and without elicitation present. This analysis used only calls from control trials (no noise present). “No Elicitation” calls were obtained from the 2 minute acclimation period at the beginning of each control trial. “Elicitation present” calls include spontaneous and elicited calls produced during the test period of control trials, when the elicitation stimuli were played at ~30s intervals. Parameters for elicitation stimuli are shown for comparison. Standard deviations are shown in parentheses.
(DOCX)

S3 Table Statistical results of one-way ANOVA tests for differences between CLCs produced with and without elicitation stimuli. No significant differences were detected.
(DOCX)

S4 Table Average call amplitudes for both call types during control (‘Base VL’) and treatment (‘Trt VL’) periods. Chirps had higher baseline amplitudes, but maximum call amplitudes were similar for both vocalization types.
(DOCX)

S5 Table Raw data from vocalizations produced during trials. F3 and F2 indicate the ratio of the power spectral density in the 3^rd^ CLC harmonic and 2^nd^ chirp harmonic to the fundamental frequencies, respectively.
(PDF)
