# Supplementary material for: Noise-Induced Frequency Modifications of Tamarin Vocalizations: Implications for Noise Compensation in Nonhuman Primates
Source: PLoS One. 2015 Jun 24;10(6):e0130211. doi: 10.1371/journal.pone.0130211 (PMC4479599; doi:10.1371/journal.pone.0130211)
Supplement: S1 Text — (DOCX) [file pone.0130211.s001.docx]

Supporting information for:

**Noise-induced frequency modifications of tamarin vocalizations: implications for noise compensation in nonhuman primates**

By Cara F. Hotchkin, Susan E. Parks, and Daniel J. Weiss

**Expanded methods**

**Data collection**

At the beginning of each session, a subject was lured from its home cage and moved to the testing area (> 100 m from the colony room), where it was allowed to enter the test cage. If the subject spontaneously produced CLCs during the acclimation period, the experimenter waited 60 seconds to determine whether the animal would continue vocalizing spontaneously. If no CLCs were produced during the one minute interval, elicitation stimuli were played at approximately 30-second intervals in order to elicit antiphonal calling from the subject. If no spontaneous CLCs were produced at all during the acclimation period, elicitation stimuli playbacks began within 15s of the end of the acclimation period, and were repeated at intervals of at least 30 seconds if the subject did not produce vocalizations.

Trials continued until the animal had produced 5 or more stereotyped CLCs, or at least 10 chirps during the test period, or until the total noise exposure (including acclimation period) reached 12 minutes. If a subject did not produce a suitable number of stereotyped CLCs or chirp vocalizations in both trials for a given day, the session was re-run on another day. At the end of a trial, the animal was returned to its home cage for a 15 – 60 minute rest period. The second trial of a session was conducted according to the same protocol counterbalancing trial type (i.e., if the first trial of a session was a control, the second trial was a treatment, and vice versa).

**Data analysis**

For all calls, duration was determined by visually inspecting a noise-reduced waveform of the call to select start and end times. Duration was calculated as the difference of these times. Spectrogram and “selection spectrum” (1024 point Hamming window, 75% overlap) views were used simultaneously to determine the minimum and maximum frequencies. Peak frequencies were determined automatically from the spectrum view, and minimum and maximum frequency measurements were chosen as inflection points on the spectrum.

**Source level of vocalizations**

Source levels for both types of vocalizations were calculated as

$$SL=RL+TL (1)$$

where RL represents the level at the receiver and TL is the transmission loss of the sound through the environment between source and receiver. Because the source in this experiment was located within 0.7 m of the microphone, TL was assumed to be negligible and excluded from further calculations.

**Call matching and effects of elicitation stimuli**

Spectro-temporal parameters of non-human primate vocalizations may vary with the context of call production. Spontaneously-produced vocalizations may have different parameters than those produced in response to another individual, and in some cases a signaler may attempt to match characteristics of the call to which it is responding [1,2], though, to the best of our knowledge, there is no evidence for this in cotton-top tamarin monkeys. However, given that CLCs may converge over time [3], we compared characteristics CLCs spontaneously produced during the acclimation period (i.e., in the absence of any elicitation stimuli), with CLCs produced following exposure to elicitation playbacks during no-noise control trials. Single-factor ANOVAs were run for each animal on three call parameters: minimum frequency, peak frequency, and duration.

**Results**

No significant differences were found between calls produced before and after exposure to elicitation stimuli (S2 and S3 Tables).


**References**

1. Sugiura H (1998) Matching of acoustic features during the vocal exchange of coo calls by Japanese macaques. Animal Behaviour 55: 673-687.

2. Candiotti A, Zuberbühler K, Lemasson A (2012) Convergence and divergence in Diana monkey vocalizations. Biology Letters 8: 382-385.

3. Weiss DJ, Garibaldi BT, Hauser MD (2001) The production and perception of long calls by cotton-top tamarins (*Saguinus oedipus*): Acoustic analyses and playback experiments. The Journal of Comparative Psychology 115: 258-271.
