# Supplementary material for: Noise-Induced Frequency Modifications of Tamarin Vocalizations: Implications for Noise Compensation in Nonhuman Primates
Source: PLoS One. 2015 Jun 24;10(6):e0130211. doi: 10.1371/journal.pone.0130211 (PMC4479599; doi:10.1371/journal.pone.0130211)
Supplement: S1 Table — Averaged over all subjects (N = 5). (DOCX) [file pone.0130211.s002.docx]

Supporting information for:

**Noise-induced frequency modifications of tamarin vocalizations: implications for noise compensation in nonhuman primates**

By Cara F. Hotchkin, Susan E. Parks, and Daniel J. Weiss

**S3 Table. Noise levels (dB re 20 µPa rms) measured during all trial sessions**. Averaged over all subjects (N=5).

|  | **Narrowband (5kHz)** | | | **Broadband (10 kHz)** | | |
| --- | --- | --- | --- | --- | --- | --- |
|  | A | B | C | D | E | F |
| **Control** | 42.5 | 42.4 | 44.4 | 42.4 | 42.4 | 42.4 |
| **Treatment** | 64.3 | 54.1 | 46.7 | 59.2 | 51.3 | 44.1 |
