# Supplementary material for: Noise-Induced Frequency Modifications of Tamarin Vocalizations: Implications for Noise Compensation in Nonhuman Primates
Source: PLoS One. 2015 Jun 24;10(6):e0130211. doi: 10.1371/journal.pone.0130211 (PMC4479599; doi:10.1371/journal.pone.0130211)
Supplement: S2 Table — This analysis used only calls from control trials (no noise present). “No Elicitation” calls were obtained from the 2 minute acclimation period at the beginning of each control trial. “Elicitation present” calls include spontaneous and elicited calls produced during the test period of control trials, when the elicitation stimuli were played at ~30s intervals. Parameters for elicitation stimuli are shown for comparison. Standard deviations are shown in parentheses. (DOCX) [file pone.0130211.s003.docx]

Supporting information for:

**Noise-induced frequency modifications of tamarin vocalizations: implications for noise compensation in nonhuman primates**

By Cara F. Hotchkin, Susan E. Parks, and Daniel J. Weiss

**S1 Table: Comparison of average minimum and peak frequencies from whole CLCs with and without elicitation present.** This analysis used only calls from control trials (no noise present). “No Elicitation” calls were obtained from the 2 minute acclimation period at the beginning of each control trial. “Elicitation present” calls include spontaneous and elicited calls produced during the test period of control trials, when the elicitation stimuli were played at ~30s intervals. Parameters for elicitation stimuli are shown for comparison. Standard deviations are shown in parentheses.

|  | **Minimum Frequency [Hz]** | | **Peak frequency [Hz]** | | **Duration [s]** | |
| --- | --- | --- | --- | --- | --- | --- |
|  | **No elicitation** | **Elicitation present** | **No elicitation** | **Elicitation present** | **No elicitation** | **Elicitation present** |
| **Bart** | 1501.3 (96.0) | 1419.4 (93.1) | 2687.0 (884.9) | 4273.9 (2237.8) | 1.86 (0.25) | 1.95 (0.34) |
| **Jerry** | 1465.8 (32.9) | 1536.8 (87.2) | 4625.0 (2021.0) | 2802.8 (1405.0) | 1.06 (1.14) | 1.78 (0.22) |
| **Mulva** | 1466.7 (128.4) | 1548.6 (96.0) | 3896.8 (971.0) | 4093.8 (765.7) | 2.48 (0.50) | 2.35 (0.32) |
| **Elicitation stimuli** | na | 1177.6 (73.5) | na | 6339.8 (1248.6) | na | 2.75 (0.13) |
