# Supplementary material for: Noise-Induced Frequency Modifications of Tamarin Vocalizations: Implications for Noise Compensation in Nonhuman Primates
Source: PLoS One. 2015 Jun 24;10(6):e0130211. doi: 10.1371/journal.pone.0130211 (PMC4479599; doi:10.1371/journal.pone.0130211)
Supplement: S3 Table — No significant differences were detected. (DOCX) [file pone.0130211.s004.docx]

Supporting information for:

**Noise-induced frequency modifications of tamarin vocalizations: implications for noise compensation in nonhuman primates**

By Cara F. Hotchkin, Susan E. Parks, and Daniel J. Weiss

**S2 Table: Statistical results of one-way ANOVA tests for differences between CLCs produced with and without elicitation stimuli.** No significant differences were detected.

|  | **Minimum Frequency [Hz]** | **Peak frequency [Hz]** | **Duration [s]** |
| --- | --- | --- | --- |
| **Bart** | F_1,6_= 4.15; p=0.09 | F_1,6_= 1.28; p=0.30 | F_1,6_= 0.74; p=0.42 |
| **Jerry** | F_1,5_= 0.98; p=0.37 | F_1,5_= 2.46 ; p=0.18 | F_1,5_= 0.88 ; p=0.39 |
| **Mulva** | F_1,7_= 1.77; p=0.22 | F_1,7_= 2.46; p=0.16 | F_1,7_= 0.40; p=0.55 |
