# Supplementary material for: Noise-Induced Frequency Modifications of Tamarin Vocalizations: Implications for Noise Compensation in Nonhuman Primates
Source: PLoS One. 2015 Jun 24;10(6):e0130211. doi: 10.1371/journal.pone.0130211 (PMC4479599; doi:10.1371/journal.pone.0130211)
Supplement: S4 Table — Chirps had higher baseline amplitudes, but maximum call amplitudes were similar for both vocalization types. (DOCX) [file pone.0130211.s005.docx]

Supporting information for:

**Noise-induced frequency modifications of tamarin vocalizations: implications for noise compensation in nonhuman primates**

By Cara F. Hotchkin, Susan E. Parks, and Daniel J. Weiss

**S4 Table. Average call amplitudes for both call types during control (‘Base VL’) and treatment (‘Trt VL’) periods.** Chirps had higher baseline amplitudes, but maximum call amplitudes were similar for both vocalization types.

|  |  | **A** | **B** | **C** | **D** | **E** | **F** |
| --- | --- | --- | --- | --- | --- | --- | --- |
| **CLCs** | **Base VL** | 56.4 | 60.5 | 61.2 | 60.8 | 61.7 | 59.9 |
|  | **Trt VL** | 68.1 | 65.4 | 64.3 | 72.1 | 68.5 | 65.1 |
|  | **Δ VL** | 11.8 | 4.9 | 3.1 | 11.3 | 6.7 | 5.2 |
| **Chirps** | **Base VL** | 64.0 | 67.1 | 65.6 | 66.1 | 67.8 | 64.9 |
|  | **Trt VL** | 68.1 | 66.5 | 67.7 | 68.6 | 69.8 | 65.5 |
|  | **Δ VL** | 4.1 | -0.5 | 2.1 | 2.5 | 2.0 | 0.6 |
