# Supplementary material for: Noise-Induced Frequency Modifications of Tamarin Vocalizations: Implications for Noise Compensation in Nonhuman Primates
Source: PLoS One. 2015 Jun 24;10(6):e0130211. doi: 10.1371/journal.pone.0130211 (PMC4479599; doi:10.1371/journal.pone.0130211)
Supplement: S5 Table — F3 and F2 indicate the ratio of the power spectral density in the 3rd CLC harmonic and 2nd chirp harmonic to the fundamental frequencies, respectively. (PDF) [file pone.0130211.s006.pdf]

Raw Data

Treatment Period Whole CLCs – Averaged for each trial

Animal	Treatment type	Bandwidth	Trial Type	Noise Level (dB)	Vocalization level (dB)	Minimum Frequency [Hz]	Peak Frequency [Hz]	Duration [s]	F3
Bart	A	5	ctrl	42.55	54.02	1482.04	5554.68	1.49	0.648705
Bart	A	5	trt	64.90	64.13	1388.50	3489.86	2.27	3.722626
Bart	B	5	ctrl	42.44	59.29	1271.30	2584.00	2.17	0.625723
Bart	B	5	trt	54.87	59.57	1387.27	4605.43	1.97	1.35179
Bart	C	5	ctrl	42.42	63.73	1360.63	3410.15	2.47	3.10062
Bart	C	5	trt	46.88	64.32	1370.13	3640.63	2.41	2.400488
Bart	D	10	ctrl	42.62	56.52	1520.70	1795.88	1.95	1.906208
Bart	D	10	trt	59.17	67.06	1382.26	4424.66	1.87	2.014575
Bart	E	10	ctrl	42.38	60.11	1478.50	7968.80	1.87	1.92094
Bart	E	10	trt	50.11	68.07	1440.88	4167.18	2.02	1.64278
Bart	F	10	ctrl	42.20	56.55	1403.48	4330.08	1.76	0.33797
Bart	F	10	trt	43.99	64.55	1294.20	3353.94	2.48	1.922318
Jerry	A	5	ctrl	42.43	41.28	1497.30	2441.43	1.43	0
Jerry	A	5	trt	64.70	59.98	1514.20	4544.90	1.97	0.954685
Jerry	B	5	ctrl	42.19	54.40	1496.15	5578.15	1.87	0.514398
Jerry	B	5	trt	54.61	55.96	1367.93	5554.70	2.02	2.053367
Jerry	C	5	ctrl	42.77	44.59	1542.80	1757.85	1.79	0
Jerry	C	5	trt	46.34	54.22	1435.63	1760.75	1.93	0.427634

Jerry	D	10	ctrl	42.63	45.89	1620.23	1929.70	1.89	0.008509
Jerry	D	10	trt	59.37	71.08	1282.24	4134.38	2.38	3.683993
Jerry	E	10	ctrl	42.42	54.94	1414.23	2727.55	2.07	0.81249
Jerry	E	10	trt	49.77	58.38	1307.08	3638.65	2.05	1.672393
Jerry	F	10	ctrl	42.35	46.65	1649.90	2381.83	1.65	0.020624
Jerry	F	10	trt	44.20	63.85	1389.71	3664.63	2.20	1.547304
Mulva	A	5	ctrl	42.64	62.63	1550.49	2835.94	2.07	0.65556
Mulva	A	5	trt	64.10	74.07	1557.36	5756.26	2.57	2.373367
Mulva	B	5	ctrl	42.56	64.75	1639.20	3975.58	2.05	0.810855
Mulva	B	5	trt	55.03	72.04	1468.99	4022.59	2.23	2.835574
Mulva	C	5	ctrl	42.21	64.83	1371.37	4187.50	2.45	2.681575
Mulva	C	5	trt	46.97	68.85	1525.64	4610.14	2.63	3.69032
Mulva	D	10	ctrl	42.31	67.71	1589.76	5238.30	2.88	1.240973
Mulva	D	10	trt	59.01	75.92	1456.06	6832.04	2.54	3.861944
Mulva	E	10	ctrl	42.24	66.42	1614.20	4222.67	2.17	1.798512
Mulva	E	10	trt	50.57	73.21	1532.05	5487.30	2.34	4.307936
Mulva	F	10	ctrl	42.65	66.35	1526.70	4103.01	2.47	1.249667
Mulva	F	10	trt	44.19	66.54	1592.78	3568.35	1.96	0.73885

Treatment Period CLC fundamental frequencies

Animal	Treatment type	Bandwidth [kHz]	Trial type	Noise level [dB]	Vocalization level [dB]	Minimum frequency [Hz]	Peak Frequency [Hz]	Duration [s]
Bart	A	5	CTRL	42.6	51.4	1511.273	1844.52	1.4856
Bart	A	5	TRT	64.9	55.8	1564.2	1725.575	2.1755
Bart	B	5	CTRL	42.4	56.8	1392.962	1713.875	2.16775
Bart	B	5	TRT	54.9	54.7	1507	1726.6	1.966667
Bart	C	5	CTRL	42.4	62.2	1422.77	1722.675	2.46575
Bart	C	5	TRT	46.9	62.6	1499.425	1761.733	2.413333
Bart	D	10	CTRL	42.6	54.6	1553.492	1795.875	1.95225
Bart	D	10	TRT	59.2	63.2	1496.908	1764.5	1.868714
Bart	E	10	CTRL	42.4	58.9	1568.48	1763.65	1.8655
Bart	E	10	TRT	50.1	66.7	1557.047	1828.14	2.0214
Bart	F	10	CTRL	42.2	52.7	1478.49	1716.8	1.76275
Bart	F	10	TRT	44.0	63.3	1409.176	1741.4	2.483
Jerry	A	5	CTRL	42.4	32.6	1579.16	1820.333	1.425
Jerry	A	5	TRT	64.7	48.4	2508.033	1898.433	1.973
Jerry	B	5	CTRL	42.2	51.9	1487.6	1798.85	1.8735
Jerry	B	5	TRT	54.6	49.1	1496.145	1760.725	2.0225
Jerry	C	5	CTRL	42.8	38.1	1535.633	1757.85	1.7905
Jerry	C	5	TRT	46.3	50.5	1505.269	1760.75	1.93275
Jerry	D	10	CTRL	42.3	40.3	1653.245	1929.7	1.89
Jerry	D	10	TRT	59.4	69.4	1417.717	2191.42	2.3784
Jerry	E	10	CTRL	42.4	52.7	1489.225	1784.175	2.066
Jerry	E	10	TRT	49.8	55.1	1443.9	1784.2	2.04675
Jerry	F	10	CTRL	42.3	41.4	1692.76	1918.95	1.6485
Jerry	F	10	TRT	44.2	61.8	1544.643	1985.486	2.197429
Mulva	A	5	CTRL	42.6	61.4	1653.42	1966.8	2.1865
Mulva	A	5	TRT	64.1	70.7	1760.833	1957.05	2.332

Mulva	B	5	CTRL	42.6	63.9	1715.773	2053.7	2.0545
Mulva	B	5	TRT	49.6	64.6	1656.474	1998.033	2.567333
Mulva	C	5	CTRL	42.2	64.0	1564.2	1843.767	2.453333
Mulva	C	5	TRT	47.0	67.9	1631.333	1999.24	2.629
Mulva	D	10	CTRL	42.3	67.0	1673.527	2010.92	2.8778
Mulva	D	10	TRT	59.0	74.3	1617.775	2055.46	2.5446
Mulva	E	10	CTRL	42.2	65.6	1671.341	1916.017	2.1725
Mulva	E	10	TRT	50.6	71.5	1597.318	2024.4	2.3355
Mulva	F	10	CTRL	42.7	65.6	1648.508	1942.4	2.470125
Mulva	F	10	TRT	44.2	65.7	1659.1	1978.517	1.964833

Treatment Period Chirps – Averaged for each trial

Animal	Treatment Type	Bandwidth	Trial Type	Noise Level [dB]	Vocalization level [dB]	Minimum frequency [Hz]	Maximum Frequency [Hz]	Peak Frequency [Hz]	Start Frequency [Hz]	Duration [s]	F2
MLHS	A	5	ctrl	42.12	66.62	6685.22	10101.71	9701.59	8852.61	0.058105	0.638997
MLHS	A	5	trt	64.11	69.52	7060.23	10446.87	10136.71	8988.13	0.061133	0.568513
MLHS	B	5	ctrl	42.45	65.28	7310.77	10143.98	9903.01	9245.08	0.058111	0.657197
MLHS	B	5	trt	55.17	67.34	7205.63	10330.29	10109.37	9409.26	0.059519	1.132152
MLHS	C	5	ctrl	42.79	65.14	7625.80	10622.17	10323.83	9669.74	0.061655	0.57625
MLHS	C	5	trt	46.49	67.14	7022.45	10070.68	9677.55	8941.79	0.058788	0.988612
MLHS	D	10	ctrl	42.30	65.30	7042.98	10271.42	9963.45	9180.88	0.063667	0.769276
MLHS	D	10	trt	58.81	68.49	7452.57	10522.46	10246.21	9369.06	0.065656	0.389831
MLHS	E	10	ctrl	42.74	66.16	7542.02	10244.00	9981.32	9460.78	0.064783	1.023865
MLHS	E	10	trt	55.08	67.64	7698.34	10466.91	10228.04	9512.76	0.076345	0.417996
MLHS	F	10	ctrl	42.18	65.17	7220.05	10133.96	9840.95	9055.67	0.0535	0.413898
MLHS	F	10	trt	44.09	64.61	7494.96	10255.68	9999.13	9192.44	0.064852	0.411544
SUS	A	5	ctrl	42.63	60.26	7642.67	10062.14	9711.73	9591.80	0.064	0.30546
SUS	A	5	trt	63.83	66.36	7873.71	10837.04	10535.13	10445.14	0.076286	0.259267
SUS	B	5	ctrl	42.39	69.69	7022.30	10877.36	10634.35	9753.93	0.069	0.454749
SUS	B	5	trt	55.34	63.28	7971.23	10951.65	10658.56	10061.70	0.059926	0.202456
SUS	C	5	ctrl	42.25	63.49	7496.13	10243.70	9878.90	9638.71	0.058667	0.678371
SUS	C	5	trt	46.64	66.83	7298.86	10598.01	10345.31	9743.40	0.0601	0.80523
SUS	D	10	ctrl	42.60	63.98	7490.01	10391.28	10159.04	9656.43	0.06081	0.550048
SUS	D	10	trt	59.47	66.06	8131.29	10977.29	10739.89	10259.12	0.067412	0.278306
SUS	E	10	ctrl	42.15	66.91	6976.52	10547.31	10249.46	9474.34	0.059	0.650069
SUS	E	10	trt	49.75	65.40	7702.12	10622.85	10375.59	9746.38	0.065	0.295092
SUS	F	10	ctrl	42.28	64.62	7141.46	10232.81	9940.43	9436.88	0.052292	0.850069
SUS	F	10	trt	44.00	66.36	7559.16	10322.10	10138.40	9883.00	0.074857	0.2821
BART	B	5	ctrl	42.44	65.47	8033.92	10588.27	10279.03	9697.43	0.055143	0.370543
BART	B	5	trt	54.87	68.23	8669.13	11734.74	11493.50	10584.56	0.064222	0.188267

BART	C	5	ctrl	42.42	67.61	8907.79	11620.56	11321.62	10634.78	0.052556	0.350722
BART	C	5	trt	46.88	69.01	8655.95	11712.77	11398.84	10544.70	0.0542	0.01011
BART	D	10	ctrl	42.62	68.38	8710.66	11665.67	11349.38	10639.16	0.05296	0.297496
BART	D	10	trt	59.17	70.73	8577.82	11379.66	11044.43	10175.54	0.058667	0.307579
BART	E	10	ctrl	42.38	69.87	9371.06	12083.39	11732.54	11041.53	0.054059	0.176453
BART	E	10	trt	50.11	73.98	8341.35	11647.96	11286.39	10614.58	0.059316	0.311495
